# Engineering the Stability of Nanozyme-Catalyzed Product for Colorimetric Logic Gate Operations

**DOI:** 10.3390/molecules26216494

**Published:** 2021-10-27

**Authors:** Lianlian Fu, Deshuai Yu, Dijuan Zou, Hao Qian, Youhui Lin

**Affiliations:** 1 College of Material Science and Engineering, Huaqiao University, Xiamen 361021, China; 20013081065@stu.hqu.edu.cn (D.Z.); hquqh@126.com (H.Q.); 2 Department of Physics, Research Institute for Biomimetics and Soft Matter, Fujian Provincial Key Laboratory for Soft Functional Materials Research, Xiamen University, Xiamen 361005, China; yudeshuai117@163.com

**Keywords:** nanozyme, logic gate, colorimetric reaction, decolorization, product stability

## Abstract

Recently, the design and development of nanozyme-based logic gates have received much attention. In this work, by engineering the stability of the nanozyme-catalyzed product, we demonstrated that the chromogenic system of 3, 3′, 5, 5′-tetramethylbenzidine (TMB) can act as a visual output signal for constructing various Boolean logic operations. Specifically, cerium oxide or ferroferric oxide-based nanozymes can catalyze the oxidation of colorless TMB to a blue color product (oxTMB). The blue-colored solution of oxTMB could become colorless by some reductants, including the reduced transition state of glucose oxidase and xanthine oxidase. As a result, by combining biocatalytic reactions, the color change of oxTMB could be controlled logically. In our logic systems, glucose oxidase, β-galactosidase, and xanthine oxidase acted as inputs, and the state of oxTMB solution was used as an output. The logic operation produced a colored solution as the readout signal, which was easily distinguished with the naked eye. More importantly, the study of such a decolorization process allows the transformation of previously designed AND and OR logic gates into NAND and NOR gates. We propose that this work may push forward the design of novel nanozyme-based biological gates and help us further understand complex physiological pathways in living systems.

## 1. Introduction

Over the past few decades, a variety of functional nanomaterials with intrinsic enzyme mimetic activities have been explored, owing to the high stability, low cost, convenient storage, and easy synthesis [1,2,3,4,5,6,7,8]. One of the extensive applications and promising developments for nanozymes is in the biocomputing system, which makes noteworthy progress in the logic gate operations that follow the laws of Boolean algebra [9,10,11,12,13,14]. Recently, Boolean algebra has been widely used in the fields of engineering technology and occupies an irreplaceable position. Additionally, according to the presence of input and output signals generated from biological molecules, the Boolean logic gates are mainly classified into AND, OR, XOR, NAND, NOR, INHIBIT, half-adder, and half-subtractor [9,15,16,17,18]. Owing to the demand for computer miniaturization, Boolean logic systems have been implemented at the molecular levels. In principle, logic gates are realized based on molecular switching or chemical reaction. Currently, the merging of nanotechnology with biology has ignited extensive research efforts for the design of logic gates and computer components based on nanozymes. Despite the tremendous opportunities and advantages, the nanozyme-based logic systems often suffer from the shortcomings of the difficulties in constructing complex logic systems, the reset of logic systems to the original state, and the limitation of the variety of nanozymes, etc. Therefore, a better understanding of the mechanism and design of the next-generation molecular logic systems has become urgent for further development.

**Figure 1:**
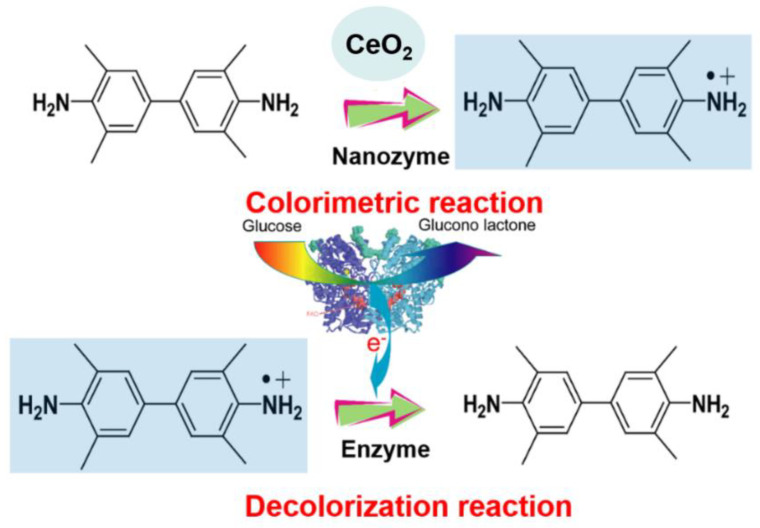
Schematic illustration for the CeO_2_-based catalyzed oxidation of TMB to yield blue oxTMB product and GOx-based decolorization.

On the other hand, great progress has been made in the construction of logic gates and biochemical computation based on DNA [19,20,21], enzymes [16,22,23,24,25], or complex biochemical systems [26]. For example, like computer devices, the use of natural enzymes for the construction of different Boolean logic systems has aroused people’s wide concern due to a huge number of enzyme-based biocatalytic processes in living organisms (plants, animals, microorganisms) [25]. Though tremendous accomplishments have been made, many of them rely on sophisticated instruments to monitor the output signals, which resulted in complicated test steps and high costs during detection. In this paper, by engineering the stability of a nanozyme-catalyzed product oxTMB through enzymatic reactions, colorimetric logic gate operations can be easily realized and output signals can be readily observed by the naked eye. In our logic systems, glucose oxidase (GOx), β-galactosidase (β-Gal), and xanthine oxidase (XO) were used as inputs, and the state of oxTMB solution was used as an output. The blue-colored solution of oxTMB obtained by the catalysis of nanozymes could become colorless by reductants, including the reduced transition state of natural enzymes (Figure 1). For instance, GOx, with flavin adenine dinucleotide (FAD) as a redox prosthetic group, is one of the typical flavin enzymes. Its biological function is to catalyze glucose to form gluconolactone while the enzyme itself is turned from GOx(FAD) to GOx(FADH2) [27,28,29]. The transition state of GOx(FADH2) can recover to GOx(FAD) again in the presence of O_2_ as indicated in Equation (1).

(1) $$\mathrm{GOx}-\left(\mathrm{FADH}2\right)+O_{2}\to \mathrm{GOx}-\left(\mathrm{FAD}\right)+H_{2}O_{2}$$

The transition state of GOx(FADH2) can also result in the reduction of oxTMB into colorless TMB (Figure 1). The obvious color change of TMB and oxTMB can be used as visual output signals. By the combination of enzymatic reactions, different colorimetric logic gate operations are easily obtained. Moreover, by taking advantage of the attractive properties of natural enzymes, simultaneous operation of several concatenated logic gates is allowed in the same chemical environment without any interference and “cross-talk” between them [25]. Furthermore, the reducibility of FADH2 is firstly introduced as a molecular switching and may provide an example for designing more complex logic systems. In light of that, we expect that this biomolecular logic system with evident and fast output signals is conducive to the area of biosensors, environmental chemistry, and therapeutics sensing.

## 2. Results

**Figure 2:**
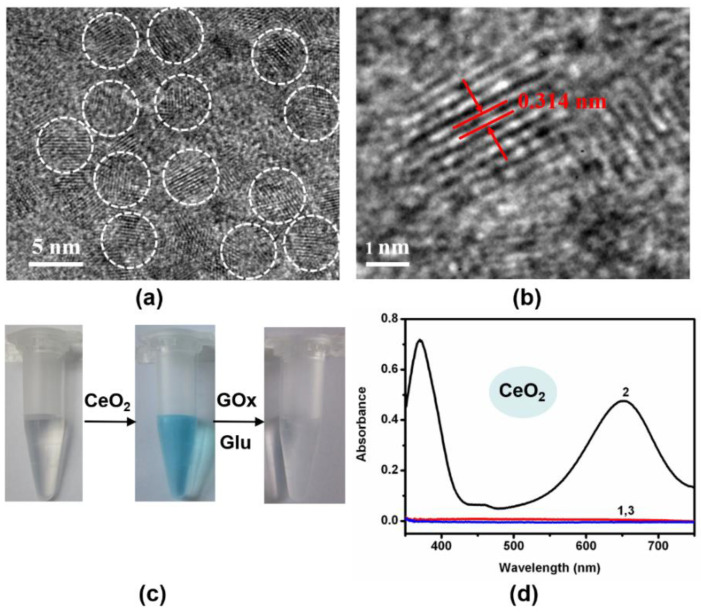
(**a**,**b**) TEM images of as-synthesized CeO_2_ NPs. (**c**) Typical photographs of TMB reaction solutions oxidized by CeO_2_ NPs and oxTMB reaction solutions reduced by GOx/glucose. (**d**) UV-vis absorption spectra of TMB reaction solutions in the presence of (1) no reactants, (2) CeO_2_ NPs, (3) CeO_2_ NPs, glucose, and GOx. ([TMB] = 120 μM, [CeO_2_ NPs] = 6 μg/mL, [glucose] = 5 mM, [GOx] = 1 unit/mL, [citrate buffer] = 40 mM, pH 6.0).

**Figure 3:**
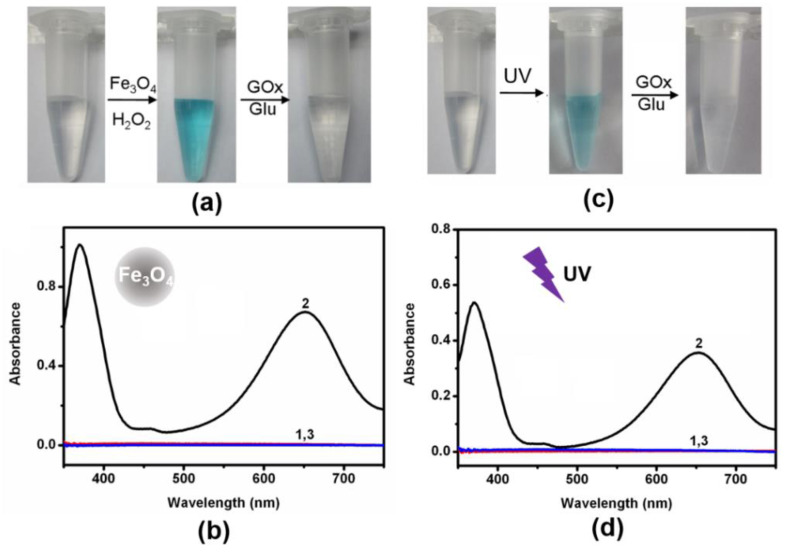
(**a**) Typical photographs of TMB reaction solutions oxidized by Fe_3_O_4_/H_2_O_2_ and oxTMB reaction solutions reduced by GOx/glucose. (**b**) UV-vis absorption spectra of TMB reaction solutions in the presence of (1) no reactants; (2) Fe_3_O_4_ NPs and H_2_O_2_; (3) Fe_3_O_4_ NPs, H_2_O_2_, glucose, and GOx. (**c**) Typical photographs of TMB reaction solutions treated by UV-light and oxTMB reaction solutions reduced by GOx/glucose. (**d**) UV-vis absorption spectra of TMB reaction solutions in the presence of (1) no reactants; (2) UV-light; and (3) UV-light, glucose, and GOx. ([TMB] = 200 μM, [Fe_3_O_4_ NPs] = 20 μg/mL, [H_2_O_2_] = 10 mM, [glucose] = 5 mM, [GOx] = 1 unit/mL, [citrate buffer] = 40 mM, pH 6.0).

**Figure 4:**
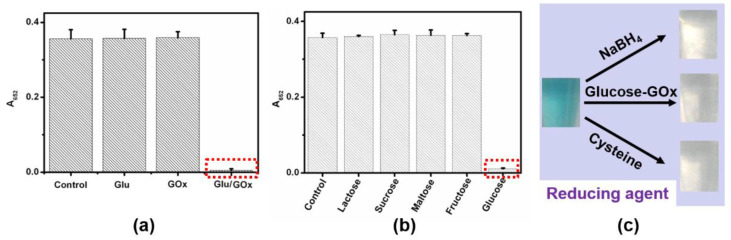
(**a**) Absorbance intensity at 652 nm of TMB reaction solutions in the presence of (from left to right): no reactants, glucose, GOx, glucose/GOx. (**b**) Absorbance intensity at 652 nm of TMB/GOx reaction solutions in the presence of (from left to right): no reactants, lactose, sucrose, maltose, fructose, and glucose. (**c**) Typical photographs of oxTMB reaction solutions reacting with NaBH_4_, glucose/GOx, and cysteine, respectively. ([TMB] = 120 μM, [CeO_2_ NPs] = 4 μg/mL, [glucose] = 5 mM, [GOx] = 1 unit/mL, [citrate buffer] = 40 mM, pH 6.0).

To implement the logic gates, CeO_2_ and Fe_3_O_4_ nanoparticles (NPs) were firstly synthesized and studied by transmission electron microscopy (TEM) imaging. The representative TEM images showed the well-dispersed CeO_2_ NPs and Fe_3_O_4_ NPs with spherical shapes were prepared (Figure 2a,b and Figure S1, Supplementary Materials). Additionally, CeO_2_ NPs with a small size and preferred plane (111) on the surface were observed (Figure S2). Additionally, the as-prepared materials were also characterized by TEM (Figure S2). The solution of the TMB substrate was almost colorless. The colorless TMB can be oxidized to a blue-colored product (oxTMB) by both CeO_2_- and Fe_3_O_4_-based nanozymes. Due to the oxidase-like activity of CeO_2_ NPs, the addition of CeO_2_ NPs into the above solution resulted in a color change from colorless to blue (Figure 2c,d). According to previous reports, Fe_3_O_4_ is a typical peroxidase mimic, which can catalyze the oxidation of TMB in the presence of H_2_O_2_ (Figure 3a,b)_._ In addition, ultraviolet light can also convert TMB to oxTMB (Figure 3c,d). It should be noted that the pH value was crucial to the oxidation reaction ofTMB. Both nanozymes showed the best catalytic performance at about pH 4.0. Since the optimal pH of natural enzymes is under physiological conditions, our logic operations for enzyme/nanozyme hybrid systems were carried out under the weak acid condition (pH 6.0). Interestingly, all the produced oxTMB solutions obtained in different ways were turned back to colorless TMB by adding glucose oxidase (GOx) and glucose (Figure 2c and Figure 3a,c). In contrast, the system containing only glucose or GOx did not show an apparent absorbance reduction toward oxTMB (Figure 4a). Furthermore, other saccharides (lactose, sucrose, maltose, and fructose) were also used to examine whether they could replace glucose. Figure 4b and Figure S4 showed, upon introduction of different saccharides into a solution containing oxTMB and GOx, no obvious color changes. We guessed that the co-existence of glucose and GOx would produce the reductive transition state (FADH2), which could reduce oxTMB to TMB. To further verify the above assumption, we added reductive substances (NaBH_4_ and cysteine) to the solutions containing oxTMB, and the same results were obtained (Figure 4c). Meanwhile, the absorption spectra of TMB reaction solutions under different conditions was obtained (Figure 2d and Figure 3b,d). Moreover, we also used another enzyme substrate 2, 2’-azinobis (3-ethylbenzthiazoline-6-sulfonic acid) diammonium salt (ABTS). As a result, both CeO_2_ NPs and Fe_3_O_4_ NPs/H_2_O_2_ systems with ultraviolet light showed a powerful ability to catalyze ABTS to produce green color, and could be further reset to their initial conditions by adding glucose/GOx (Figure S5). Taking the reversible optical response of TMB into account, the colorless solution and blue solution could be defined as OFF and ON states, respectively.

**Figure 5:**
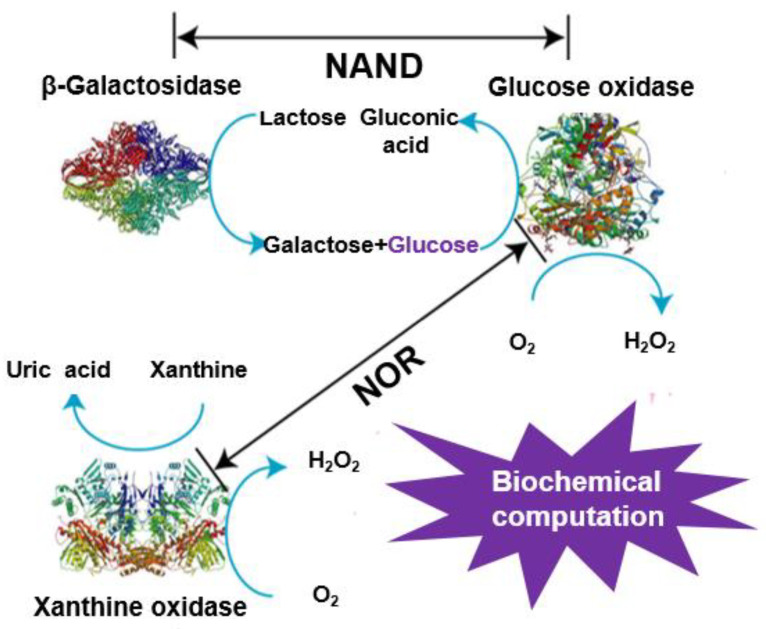
The operation of logic gates based on enzymatic reactions.

**Figure 6:**
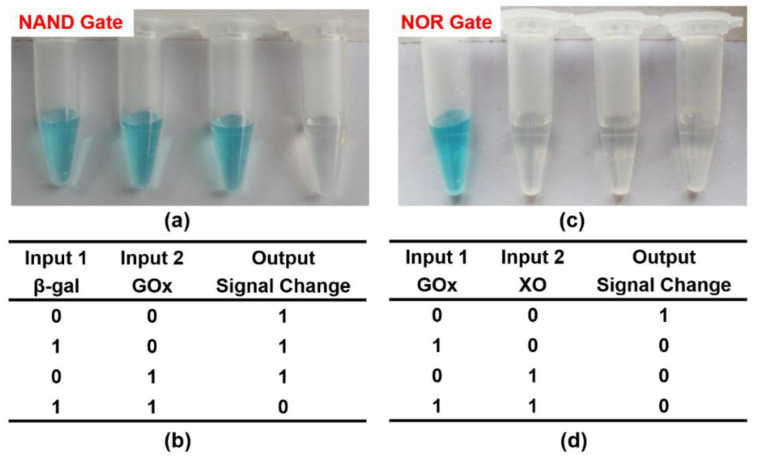
Output results and truth table of different logic gates: (**a**,**b**) NAND, (**c**,**d**) NOR. ([TMB] = 120 μM, [CeO_2_ NPs] = 4 μg/mL, [lactose] = 25 mM, [glucose] = 5mM, [β-gal] = 10 units/ mL, [GOx] = 1 unit/mL, [xanthine] = 100 μM, [XO] = 0.04 unit/mL, [citrate buffer] = 40 mM, pH 6.0).

The above results certified that the state of oxTMB was dependent on the participation of O_2_^−^ and FADH2. Inspired by the above phenomena, we firstly constructed an NAND gate by employing two enzymes: β-gal and GOx as inputs, and the final color of the reaction products as outputs (Figure 5). The initial system contained lactose, CeO_2_ NPs, TMB, and oxygen in citrate buffer (pH 6.0). β-gal could hydrolyze lactose to generate galactose and glucose. GOx presented glucose-oxidase activity, through which glucose could be catalyzed to produce gluconic acid, H_2_O_2_, and FADH2. Only when both enzymes participated (input = 1/1) could FADH2 be generated by the cascade reaction, thus simulating the logic operation NAND. Figure 6a,b displayed the corresponding output results with different combinations of the input signals. Obviously, the formation of FADH2 could not be achieved when either or both of the inputs were absent (input = 0/0, 1/0, 0/1). As a result, CeO_2_ NPs successfully catalyzed the oxidation of TMB to generate a blue color product (output = 1). Besides, in the presence of both β-gal and GOx (input = 1/1), the enzymatic cascade reactions could take place smoothly, successfully producing FADH2. Thus, the system displayed a color change from blue to colorless (output = 0). This result correlated well with the proper execution of the NAND logic operation.

Analogously, an NOR logic gate was constructed that used GOx and XO as inputs and an intense blue solution as the output signal (Figure 5). The starting solutions containing glucose, xanthine, CeO_2_ NPs, TMB, and oxygen in citrate buffer (pH 6.0) were first added to four tubes. Then, input-1 (GOx) and input-2 (XO) were added according to their corresponding states for each logic operation (0/0, 1/0, 0/1, 1/1). FADH2 can be generated in two parallel catalytic reactions, thus achieving the NOR logic gate. In the absence of any input, the logic operation produced a visual signal (oxTMB with blue color) that is recognized by the naked eye (input = 1). For another, FADH2 was generated when either or both inputs were added. As a result, the formation of oxTMB could be effectively prevented due to the reduction properties of FADH2, and the mixture became colorless (output = 0) (Figure 6c,d). This response from the solution corresponds to the NOR logic gate.

**Figure 7:**
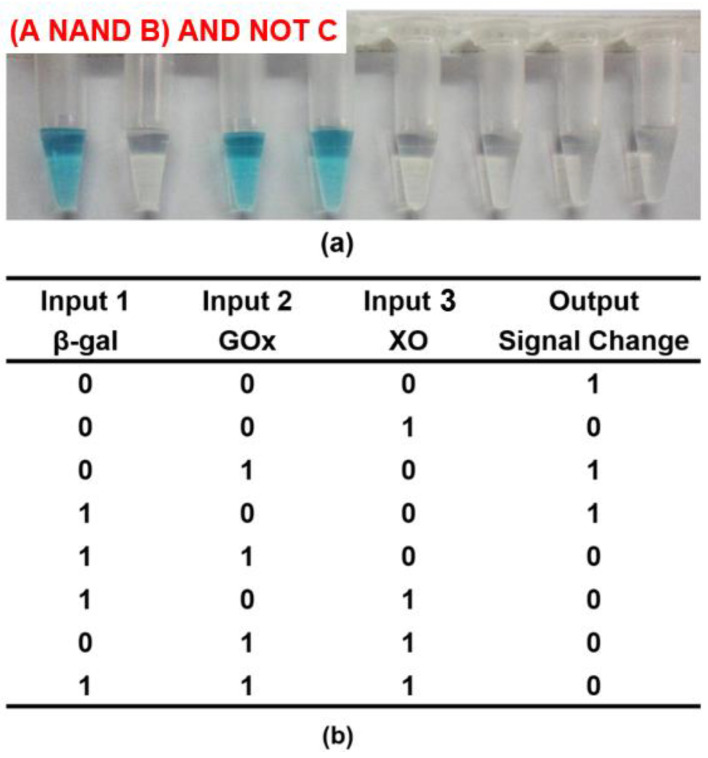
(**a**) Output result, and (**b**) truth table of (A NAND B) AND NOT C logic gates. ([TMB] = 120 μM, [CeO_2_ NPs] = 4 μg/mL, [β-gal] = 10 units/mL, [GOx] = 1 unit/mL, [lactose] = 25 mM, [glucose] = 5mM, [xanthine] = 100 μM, [XO] = 0.04 unit/mL, [citrate buffer] = 40 mM, pH 6.0).

Except for the simple logic circuit, the multi-enzyme-based complex system was also explored. Based on the above NAND and NOR gates, we further demonstrated the operation of a Boolean logic system to calculate (A NAND B) AND NOT C by employing three enzyme inputs (β-gal, GOx, and XO). The starting solutions contained lactose, xanthine, oxygen, TMB, and CeO_2_ NPs in citrate buffer (pH 6.0). FADH2 could be generated when both β-gal and GOx co-existed. Specifically, the reaction process was as follows: β-gal could firstly hydrolyze lactose to generate galactose and glucose. GOx presented glucose-oxidase activity, through which the intermediated state of GOx(FADH2) could be produced during the oxidation of glucose. In addition, FADH2 could also be produced by introducing XO, since xanthine could be catalyzed by XO to generate FADH2. As a result, the formation of oxTMB could be effectively prevented due to the reduction properties of FADH2, and the system displayed a color change from blue to colorless (output = 0). As shown in Figure 7, when input 1 and input 2 did not exist together or input 3 was absent, FADH2 could not be produced to prevent the formation of oxTMB (output = 1). This result correlated well with the (A NAND B) AND NOT C logic gate.

## 3. Discussion

It is well-known that a bi-enzymatic (horseradish peroxidase, HRP and GOx) cascade is used for the oxidation of TMB into the blue product oxTMB, which has been widely used in sensing applications [27]. Amazingly, we found that oxTMB was not stable and the blue color can fade easily in the presence of reductants, including the reduced transition state of glucose oxidase and xanthine oxidase. It means that a false-negative test result might occur with the use of HPR-GOx for sensing applications. During the sensing process, the oxygen should be sufficient, which ensures that the transition state of GOx(FADH2) can quickly recover to Gox(FAD). In addition, the study of such a decolorization process allows us to construct different logic gates by combining enzymatic reactions. More importantly, since the output signal is 1 (the color of solution is blue) in the initial state, such a decolorization process can transform previously designed AND and OR logic gates into NAND and NOR gates.

In this work, we found that oxTMB can be reduced by FADH2. By taking advantage of this phenomenon, we combine the reducibility of FADH2 with the catalytic activity of nanozymes to achieve logic functions, which has not been reported before. Furthermore, since catalytic reactions can work together smoothly, this system can produce a multi-input network without any interference. More importantly, this process can be easily monitored by UV-vis spectroscopy and even by the naked eye, which might dramatically improve the stability of logical operations. Combined with these distinct advantages, we expect that our work will be beneficial in future biomedical and biological applications.

## 4. Materials and Methods

### 4.1. Materials

Cerium nitrate hexahydrate (Ce(NO_3_)_3_·6H_2_O) and ammonium hydroxide (25–28 wt%, NH_3_·H_2_O) were obtained from Sinopharm Chemical Reagent Co. (Shanghai, China). Sodium hydroxide, 3,3,5,5-Tetramethylbenzidine, 1,3,5-trimethylbenzene, 2,2’-azinobis (3-ethylbenzthiazoline-6-sulfonic acid) diammonium salt, glucose oxidase (EC 232-601-0), xanthine (EC 200-718-6), β-galactosidase (EC 232-864-1), and xanthine oxidase (EC 32-657-6) were purchased from Sigma-Aldrich. Glucose and lactose were obtained from Aladdin Co. (Shanghai, China). H_2_O_2_ was obtained from Beijing Chemicals (Beijing, China). All other reagents were of analytical reagent grade and used as received. Ultrapure water (18.2 MU; Millipore Co., Burlington, MA, USA) was used throughout the experiment.

### 4.2. Measurements and Characterizations

Transmission electron microscope images were collected by a high-resolution TECNAI F30 HRTEM operated at 200 kV. The crystalline structure of the as-prepared CeO_2_ NPs was measured by a Bruker AXS D8 advance X-ray diffractometer with Cu-Kα radiation. The particle sizes of CeO_2_ NPs were measured by using a NanoBrook Omni instrument. The UV-Vis absorption spectra were recorded using a JASCO V-550 UV/Visible spectrophotometer (JASCO International Co., LTD., Tokyo, Japan).

### 4.3. Synthesis of CeO_2_ NPs and Fe_3_O_4_ NPs

CeO_2_ NPs were prepared based on a previously reported procedure [30]. Briefly, 2.17 g of cerium (III) nitrate were first dissolved in 5.0 mL of pure water, followed by mixing with 1.0 M dextran. Next, the above mixture was dropped into 30.0 mL of ammonium hydroxide solution (25%) and stirring was continued. The mixture was continually stirred at room temperature for 24 h. Moreover, the suspension was centrifuged. Finally, the resultant precipitate was washed with distilled water three times and then dissolved in 40 mL of distilled water. Fe_3_O_4_ NPs were synthesized as reported previously [31]. In brief, 4.0 mmol of FeCl_3_ and 0.68 mmol of trisodium citrate were mixed in 20.0 mL of ethylene glycol, followed by the addition of 1.20 g of sodium acetate and stirred for 30 min. Then, the mixture was transferred to a Teflon-lined stainless steel autoclave tube, which was heated at 200 °C and maintained for 10 h. The black products indicated the formation of Fe_3_O_4_ nanoparticles, which were washed with ethanol and deionized water several times before further use.

### 4.4. CeO_2_ and Fe_3_O_4_ NPs-Based Logic Systems in Solution

Firstly, the oxidase-like activities of CeO_2_ NPs were studied by incubating 1 mL of solution of the 40 mM citrate buffer (pH 6.0), 3 μL of 40 mM TMB, and 1.5 μL of 4 mg/mL CeO_2_ NPs suspension at 35 °C for 15 min. Similarly, the peroxidase-like activities of Fe_3_O_4_ NPs were investigated by incubating 1 mL of solution of the 40 mM citrate buffer (pH 6.0), 5 μL of 40 mM TMB, 10 μL of 2 mg/mL Fe_3_O_4_ NPs suspension, and 20 μL of 500 mM H_2_O_2_ at 35 °C for 15 min. Moreover, the effects of 254 nm ultraviolet light on oxidation of TMB were observed for 1 mL of solution of the 40 mM citrate buffer (pH 6.0) and 5 μL of 40 mM TMB under the radiation of the UV lamp at 35 °C for 20 min. Afterwards, both the 10 μL of 500 mM glucose and 50 μL of 20 units/mL GOx dissolved oxygen in equilibrium with air were added in the previous solutions, and the color turned from blue to colorless. Additionally, no color fading was observed in the solutions with the addition of either glucose or GOx. The influences of pH from 4.0 to 6.0 in the 1-mL solutions on the oxidase-like activities of ultraviolet light (40 mM citrate buffer, 3 μL of 40 mM TMB), CeO_2_ NPs (40 mM citrate buffer, 3 μL of 40 mM TMB, and 0.5 μL of 4 mg/mL CeO_2_ NPs suspension), and peroxidase-like activities Fe_3_O_4_ NPs (40 mM citrate buffer, 3 μL of 40 mM TMB, 2 μL of 2 mg/mL Fe_3_O_4_ NPs suspension, and 10 μL of 500 mM H_2_O_2_) were also discussed. In addition, the various saccharides including lactose, sucrose, maltose, and fructose were evaluated on the appearance properties of oxTMB/GOx reaction solutions. The UV-vis absorption spectra and corresponding optical photographs of each input were recorded.

Next, the oxidase-like activities of ultraviolet light, CeO_2_ NPs, and oxidase-like activities of Fe_3_O_4_ NPs were investigated on another enzyme-substrate ABTS (40 mM) in the citrate buffer (pH 6.0). All the colors of 1-mL solutions oxidized by UV-light (40 mM citrate buffer and 200 μL ABTS), with CeO_2_ NPs (40 mM citrate buffer, 200 μL ABTS, and 8 μL of 4 mg/mL CeO_2_ NPs suspension), and Fe_3_O_4_ NPs (40 mM citrate buffer, 500 μL ABTS, 20 μL of 2 mg/mL Fe_3_O_4_ NPs suspension, and 100 μL of 500 mM H_2_O_2_) incubated at 35 °C for 15 min turned to green. Additionally, the ABTS^•+^ reaction solutions were reduced with the addition of 200 μL of 20 units/mL GOx and 40 μL of 500 mM glucose solutions.

According to the phenomenon, the NAND logic gate could be constructed. Different input combinations of 100 μL of β-gal (input-1, 100 units/mL) and 50 μL of GOx (input-2, 20 units/mL) (0/0, 1/0, 0/1, 1/1) were added into four Eppendorf tubes with the 1-mL starting solutions (3 μL of 40 mM TMB, 100 μL of 250 mM lactose, 1 μL of 4 mg/mL CeO_2_ NPs, and oxygen dissolved in the 40 mM citrate buffer, pH 6.0). Each mixture was incubated at 35 °C for 15 min. To perform the NOR logic gate, 50 μL of GOx (input-1, 20 units/mL) and 4 μL of XO (input-2, 10 units/mL) were combined in all possible ways and added to four starting 1-mL solutions (3 μL of 40 mM TMB, 1 μL of 4 mg/mL CeO_2_ NPs, 10 μL of 500 mM glucose, 2 μL of 50 mM xanthine, and oxygen dissolved in 40 mM citrate buffer, pH 6.0). Each mixture was incubated at 35 °C for 15 min. Above all, to perform a complex [(A NAND B) AND NOT C] system, 100 μL of β-gal (input-1, 100 units/mL), 50 μL of GOx (input-2, 20 units/mL), and 4 μL of XO (input-3, 10 units/mL) were combined in all possible ways and added to eight equal starting solutions (3 μL of 40 mM TMB and 1 μL of 4 mg/mLCeO_2_ NPs, 100 μL of 250 mM lactose, 10 μL of 500 mM glucose, 2 μL of 50 mM xanthine, oxygen, dissolved in the 40 mM citrate buffer, pH 6.0). Finally, the UV-vis absorption spectra and corresponding optical photographs of each input were recorded.

## 5. Conclusions

In summary, we successfully demonstrated that CeO_2_ NPs could catalyze the oxidation of TMB to generate a blue color product and the system could be easily reset in the presence of FADH2 without any complicated procedures. Furthermore, we constructed NAND, NOR logic gates, and a logic network [(A NAND B) AND NOT C] when using enzymes (GOx, β-gal or XO) as inputs and the state of oxTMB as the output. Notably, we combined nanozymes with natural enzymes to achieve logic functions. Such logic gates are easily controllable, low cost, and label-free. The distinctive advantage of this logic system is that output signals could be monitored by UV-vis absorption spectra and even the naked eye. We expect that this work will be highly beneficial in molecular computers and pave the way for tangible applications of various Boolean logic systems in the future.

## Data Availability

Data is provided within the article and supplementary material.

## Supplementary Materials

The following are available online, Figure S1: TEM image of Fe_3_O_4_ NPs. Figure S2: (a) Wide-angle powder XRD pattern and (b) size distribution of the CeO_2_ NPs. Figure S3: Absorbance intensity at 652 nm of TMB reaction solutions treated by (a) UV-light, (b) CeO_2_ NPs, and (c) Fe_3_O_4_ NPs and H_2_O_2_ under different pH. Figure S4: Effect of various saccharides on the appearance properties of oxTMB/GOx reaction solutions: (1) control, (2) lactose, (3) sucrose, (4) maltose, (5) fructose, (6) glucose. Figure S5: Typical photographs of ABTS reaction solutions oxidized by (a) UV-light, (b) CeO_2_, (c) Fe_3_O_4_/H_2_O_2_, and ABTS^•+^ reaction solutions reduced by GOx/glucose.

## Sample Availability

Samples of the compounds are not available from the authors.
