# Supplementary material for: Engineering the Stability of Nanozyme-Catalyzed Product for Colorimetric Logic Gate Operations
Source: Molecules. 2021 Oct 27;26(21):6494. doi: 10.3390/molecules26216494 (PMC8587802; doi:10.3390/molecules26216494)
Supplement: Supplementary file 1 [file molecules-26-06494-s001.zip › molecules-1408520-supplementary.pdf]

Supplementary Material

Engineering the Stability of Nanozyme-catalyzed Product for Colorimetric Logic Gate Operations

Lianlian Fu [1,*,†], Deshuai Yu [2,†], Dijuan Zou [1], Hao Qian [1] and Youhui Lin [2,*]

1 College of Material Science and Engineering, Huaqiao University, Xiamen 361021, China; 20013081065@stu.hqu.edu.cn (D.Z.); hquqh@126.com (H.Q.)
2 Department of Physics, Research Institute for Biomimetics and Soft Matter, Fujian Provincial Key Laboratory for Soft Functional Materials Research, Xiamen University, Xiamen 361005, China; yudeshuai117@163.com (D.Y.)
* Correspondence: fll@hqu.edu.cn (L.F.); linyouhui@xmu.edu.cn (Y.L.)
† These authors contributed equally to this work.

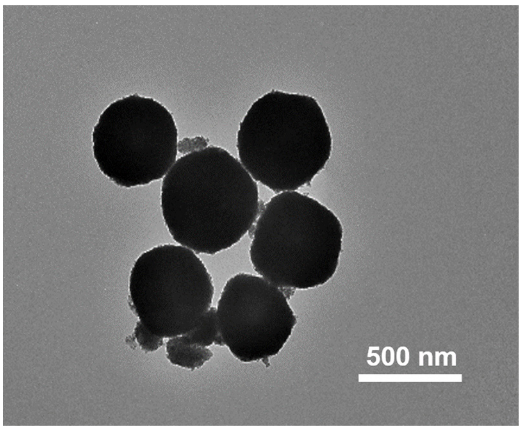

Figure S1. TEM image of Fe_3O_4 NPs.

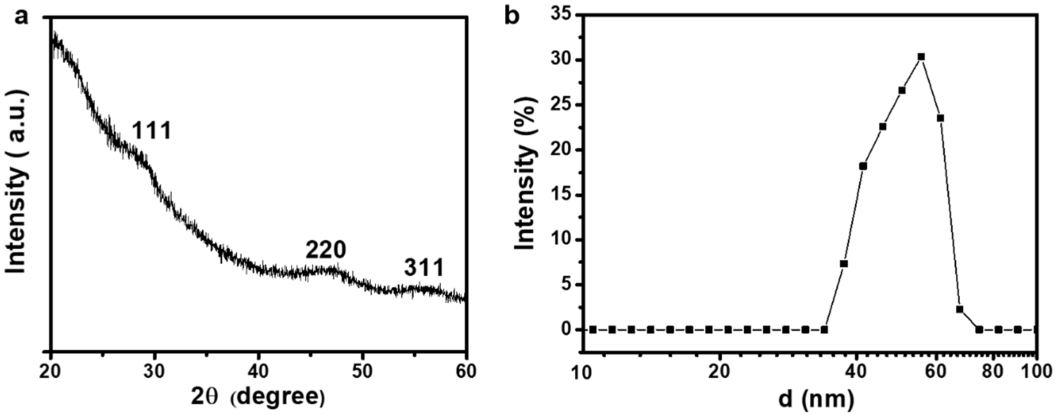

Figure S2. (a) Wide-angle powder XRD pattern and (b) size distribution of the CeO_2 NPs.

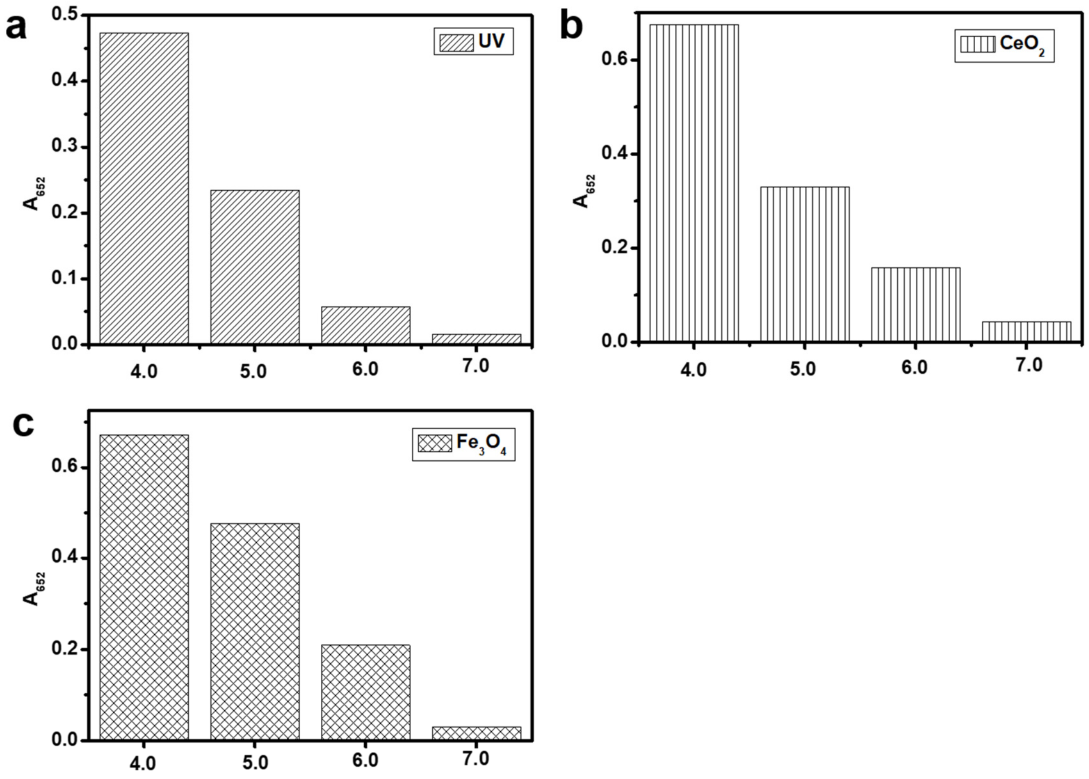

Figure S3. Absorbance intensity at 652 nm of TMB reaction solutions at 35 °C for 15 min treated by (a) UV-light, (b) CeO_2 NPs, and (c) Fe_3O_4 NPs and H_2O_2 under different pH. ([TMB] = 120 μM, [CeO_2 NPs] = 2 μg/mL, [Fe_3O_4 NPs] = 4 μg/mL, [H_2O_2] = 5 mM, [citrate buffer] = 40 mM).

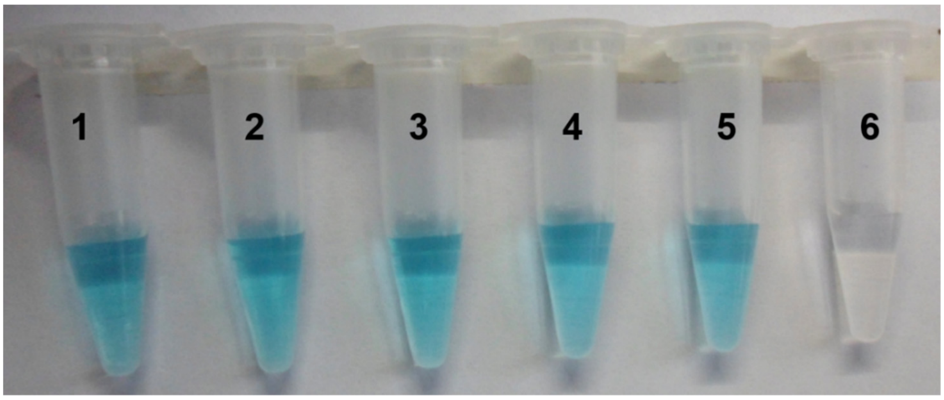

Figure S4. Effect of various saccharides on the appearance properties of oxTMB/GOx reaction solutions at 35 °C for 15 min (from left to right): (1) control, (2) lactose, (3) sucrose, (4) maltose, (5) fructose, (6) glucose. ([TMB] = 120 μM, [CeO_2 NPs] = 4 μg/mL, [GOx] = 1 unit/mL, [glucose] = 5 mM, [citrate buffer] = 40 mM, pH 6.0).

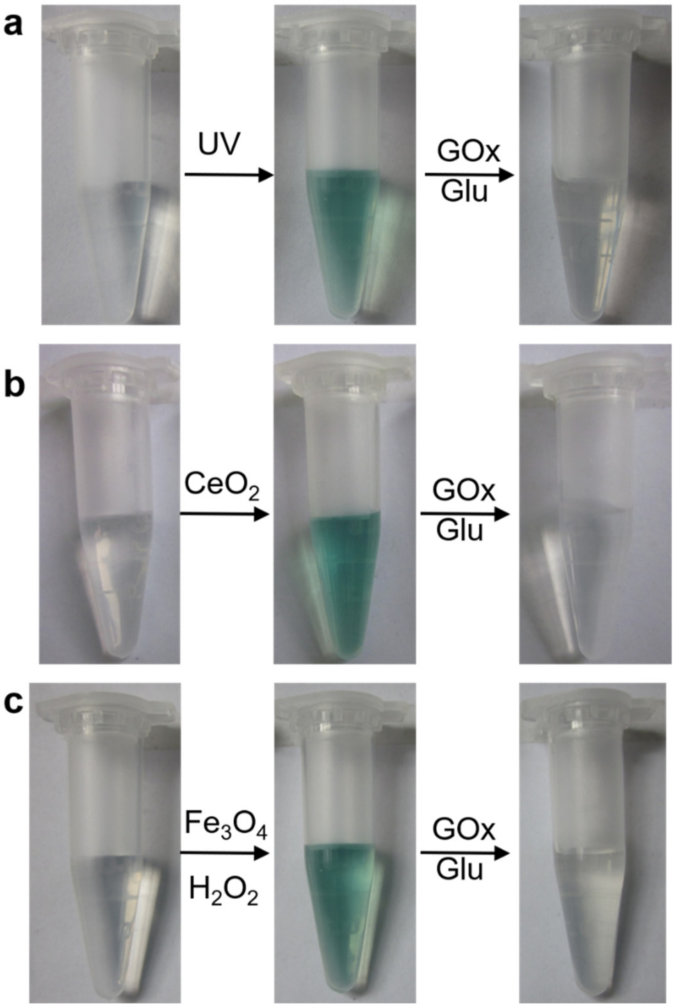

Figure S5. Typical photographs of ABTS reaction solutions at 35 °C for 15 min oxidized by (a) UV-light, ([ABTS] = 8 mM) (b) CeO_2, ([ABTS] = 8 mM, [CeO_2 NPs] = 32 μg/mL) (c) Fe_3O_4/H_2O_2, and $ABTS^{\bullet+}$ reaction solutions reduced by Gox/glucose. ([ABTS] = 20 mM, [Fe_3O_4 NPs] = 40 μg/mL, [H_2O_2] = 50 mM, [GOx] = 4 units/mL, [glucose] = 20 mM, [citrate buffer] = 40 mM, pH 6.0).
